# Habituation of exploratory behaviour in VPA rats: animal model of autism

**DOI:** 10.2478/intox-2013-0033

**Published:** 2013-12

**Authors:** Lucia Olexová, Tomáš Senko, Peter Štefánik, Alžbeta Talarovičová, Lucia Kršková

**Affiliations:** 1 Department of Animal Physiology and Ethology, Faculty of Natural Sciences, Comenius University in Bratislava, Bratislava, Slovakia; 2 Neuroendocrinology Group, Faculty of Mathematics and Natural Sciences, University of Groningen, Groningen, The Netherlands

**Keywords:** autism, animal model, VPA rats, exploratory behaviour, habituation

## Abstract

Autism is a neurodevelopmental disorder with multifactorial aetiology, represented as impairment in social behaviour, communication and the occurrence of repetitive activities, which can be observed in the early life. The core features are frequently accompanied by other manifestations, including limited environmental exploration. The aim of the presented study, realised on an animal model of autism – VPA rats, *i.e.* animals prenatally affected with valproic acid on gestation day 12.5, was to investigate the habituation process of exploratory activity (manifested by a gradual decrease in the intensity of locomotor activity), which reflects the stage of the central nervous system. VPA rats were tested in open-field in three developmental periods – weaning (postnatal day 21 – PND 21), puberty (PND 42) and adulthood (PND 72). In each period of ontogenesis, the rapidity of habituation was evaluated by using the method of linear regression. Compared to controls, VPA rats showed a significant decrease in the intensity and an increase in the rapidity of exploratory activity habituation during puberty and adulthood. Our results indicate that the animal model of autism, *i.e.* VPA rats, showed disabilities in the development of the nervous system. These findings can help confirm not only the validity of this animal model of autism but can also help better understand neuronal changes in humans with autism.

## Introduction

Autism is a neurodevelopmental disorder with multifactorial aetiology characterised by severe deficits in social reciprocity and communication and an occurrence of repetitive behaviour (American Psychiatric Association, 2000). This disorder is usually diagnosed by three years of age (Levy *et al.,* 2009), but video analyses of infants diagnosed later with autism have revealed disruptions in development already by the first year of life (Teitelbaum *et al.,* 1998; Baranek, 1999; Osterling *et al.,* 2002; Esposito, 2011). The core manifestations of autism are frequently accompanied by other features, *e.g.* hyperactivity (Canitano & Scandura, 2011), sleep disturbances, obsessive-compulsive behaviour (Amaral & Corbett, 2003), aberrant sensitivity to sensory stimulation (Militerni *et al.,* 2000), anxiety (Groden *et al.,* 1994; Gillott *et al.,* 2001; Towbin *et al.,* 2005), decrease of motivation (American Psychiatric Association, 2000) and delayed motor development (Ohta *et. al.,* 1987; Landa & Garrett-Mayer, 2006; Provost *et al.,* 2007; Downey & Raport, 2012; Flanagan *et al.,* 2012). However studies on autism focused primarily on social deficit. According to Kawa and Pisula (2010), only the analysis of behaviour not directly linked with social interaction may provide new important information about autism.

Recently, some authors have explored links between autism and limited environmental exploration. Children with autism spent significantly less time by active exploration than normal children (O'Neil & Happé, 2000; Pierce & Courchesne, 2001).

Moreover, animal models of autism, such as rats prenatally exposed to valproic acid (VPA rats) (Schneider & Przewłocki, 2005; Schneider *et al.*, 2006; Kerr *et al.,* 2013), BALB/cByJ (Moy *et al.,* 2008), *Pax6* heterozygous mutant (*rSey* ^*2*^/+) rats (Umeda *et al.,* 2010), and mice with disruption in chromosome 7 (analogue of human locus 15q11-13) (Tamada *et al.,* 2010), showed, in addition to other typical autistic behavioural deficits, also a reduction of exploratory behaviour.

The observed changes in exploratory behaviour are strongly connected with alterations in particular brain areas. Changes in the number of Purkinje cells in cerebellar vermal lobules have been observed in both VPA rats (Rodier *et al.,* 1996, 1997) and autistic patient (Pierce & Courchesne, 2001). The decreased number of Purkinje cells correlated with reduced environmental exploration (Pierce & Courchesne, 2001). Alteration in structures involved in regulation of fear processing can provide a further explanation of reduced exploratory behaviour and similar, changes have been observed in VPA rats (Markram *et al.,* 2008; Rinaldi *et al.,* 2008; Sui & Chen, 2012) as well as in autistic patients (Schumann *et al.,* 2004; Bachevalier & Loveland 2006).

The aim of the presented study was to investigate the habituation process of exploratory behaviour of VPA rats (manifested by a gradual decrease in the intensity of locomotor activity in open-field). This process is known to reflect a stage of the development of the central nervous system (CNS) (Morokuma *et al.,* 2004) and is changing during development (Parsons *et al.,* 1973; Chapillon & Roullet, 1997; Lynn & Brown, 2009).

Habituation is defined as “the decrement in response following repeated stimulation with the same stimulus” and it is considered to be a basic form of learning (Morokuma *et al.,* 2004). In rodents, habituation is commonly evaluated as a quantitative decrease in exploratory behaviour in response to continued or repeated exposure to an unknown environment (Leussis & Bolivar, 2006).

Impaired habituation has been reported in individuals with Down's syndrome (Hepper & Shahidullah, 1992), schizophrenia (Ludewig *et al.,* 2003), mental retardation (Gandhavadi & Melvin, 1985), depression (Michael *et al.,* 2004), epilepsy (Rogozea & Florea-Ciocoiu, 1982), and Alzheimer's disease (Vanini *et al.,* 2010).

Impaired neural habituation has been reported in people with autism (Kleinhans *et al.,* 2009). Toddlers with more severe features of autism showed slower habituation to faces (Webb *et al.,* 2010).

Information about changes of the VPA rat habituation process during ontogenesis can help to confirm not only the validity of this animal model of autism but also contribute to a better understanding of changes in CNS functional development in humans with autism.

## Methods

### Animals

Wistar rats (200–300 g, Institute of Experimental Pharmacology and Toxicology, Dobra Voda, SR) were housed in groups of two or three animals in standard light conditions (12:12; lights on at 6 a.m.) with food (Dos-2b OVO, Dobra Voda, SR) and water *ad libitum*. After an acclimatisation period of 7 days, male and female rats were mated overnight and the presence of spermatozoa in vaginal smear was considered the first day of gestation (GD 1) (Schneider & Przewłocki, 2005; Schneider *et al.,* 2006). On GD 12.5 (in the middle of the light phase), half of the females received a single intraperitoneal injection of sodium valproate (VPA; Sigma, USA) dissolved in saline (pH = 7.3; 250 mg/ml) in the dose of 600 mg/kg. The other females served as a controls (C) and received saline (pH = 7.3; 250 mg/ml) at the same time of gestation (Schneider & Przewłocki, 2005; Schneider *et al.,* 2006). Females were housed in groups of two or three animals until GD 20, later they were housed individually.

After delivery, valproate-treated (VPA) and control (C) females were allowed to raise their offspring until weaning on postnatal day (PND) 21. On PND 1, the litters were culled to 8 animals per litter (4 males, 4 females). The other animals of the litter were used for sample preparation for further analysis. After weaning, the rats of either sex were housed separately in groups of 4 animals per cage.

The offspring of VPA (n=18; 9 males, 9 females) and C (n=18; 9 males, 9 females) were tested in the open-field test in three developmental periods: weaning (PND 21), puberty (PND 42) and adulthood (PND 72).

### Behavioural test

Spontaneous exploratory behaviour (locomotor activity) and its habituation were evaluated in an open-field test. All animals (VPA and C) were tested in Conducta (Experimetria Ltd., Hungary), a system used the continuous recording of kinetic activity of laboratory rodents. The testing chamber of this system consisted of a dark plastic box (48×48×40 cm) with its floor divided into 25 squares and built-in infrared beam lights (16 diodes at 16 mm distance from each other in the three lines) for recording the animals’ movement.

During the light phase (12:00–18:00 h), each animal was put into the centre of the testing chamber and subsequently tested for 20 minutes. The information about locomotor activity (horizontal + vertical), expressed as a number of beam breaks during horizontal and vertical movement, were exported from Conducta system in four five-minute intervals (the first interval: 0–5 min; the second interval: 5–10 min, the third interval: 10–15 min and the fourth interval: 15–20 min). A gradual decrease of locomotor activity during the test represented an uninterrupted habituation.

### Statistical analysis

The habituation process was evaluated using the exponential function Y (t) = Y_0_.e^–kt^ (Y = the amount of locomotor activity in the individual five minute interval, *k* = the individual rate of habituation) was used as a model of the habituation course of locomotor activity (Dubovický *et al.,* 1999).

The intensity of locomotor activity (for the whole duration of the test) and k-value of habituation of VPA and C animals were analysed using STATISTICA v 7.0 (StatSoft, Inc., Tulsa, USA) and repeated measures analysis of variance (ANOVA) with the fixed effects of group, sex, and age and their interaction. The effect of litter nested within the group was included in the model. If the interaction was significant, the differences between individual groups were evaluated by Fisher LSD *post hoc* test. The results are expressed as means ± SEM.

### Ethics statement

The methods and procedures of the present study were approved by the local Ethical Committee of the Comenius University in Bratislava, Slovak Republic and the Directive of the European Parliament and of the Council on the protection of animals used for scientific purposes (2010/63/EU) was followed. All efforts were made to minimise the number of animals used and their suffering.

## Results

**Figure 1:**
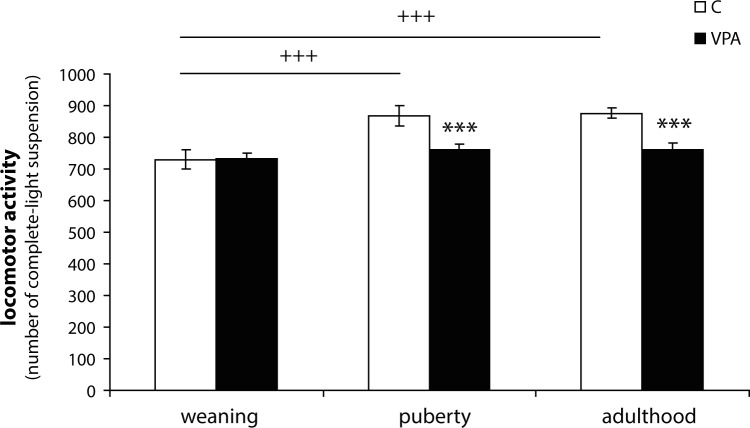
Locomotor activity of control (C, n=18) and valproic (VPA, n=18) rats in the open-field test. Data are given as means ± SEM per 20 min. Asterisks indicate significant differences between C and VPA group (*** *p<*0.001), plus they indicate significant differences between ontogenetic stages in the C group (+++ *p<*0.001).

A statistical analysis of locomotor activity has revealed a significant effect of the group (F_1,28_=19.391; *p<*0.001), litter (nested within the group) (F_4,28_=6.182; *p<*0.01), age (F_2,56_=10.972; *p<*0.001), and interaction age*group (F_2,56_=4.622; *p<*0.05). VPA rats showed decreased locomotor activity compared to the control group in puberty (*p<*0.001) and in adulthood (*p<*0.001) (Figure 1). In weaning, there were no significant differences between VPA and C rats. Sex differences were not found at any stage of ontogenesis. In the VPA group, there were no significant differences in locomotor activity among the three ontogenetic stages. We observed changes in the developmental period only in C animals. These animals showed an increase of locomotor activity in puberty (*p<*0.001) and adulthood (*p<*0.001), compared to the weaning period (Figure 1).

**Figure 2:**
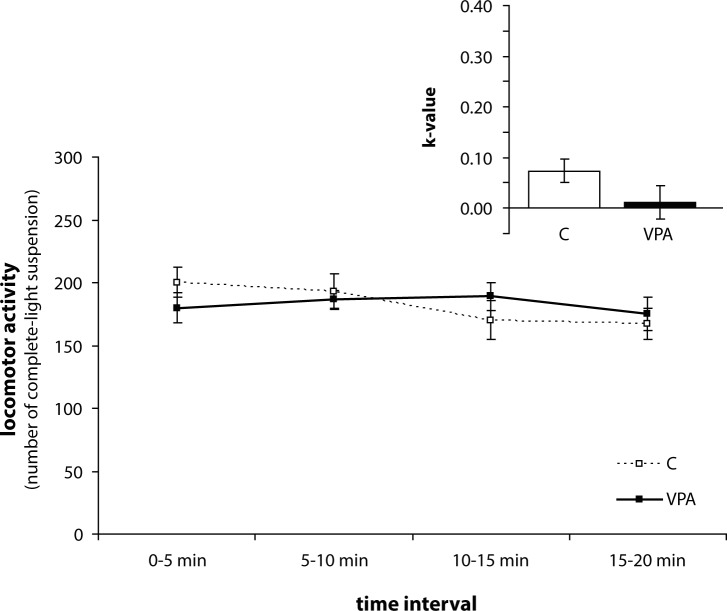
Habituation dynamics and rate (k-value) of control (C, n=18) and valproic (VPA, n=18) rats during weaning. Data are given as means ± SEM.

**Figure 3:**
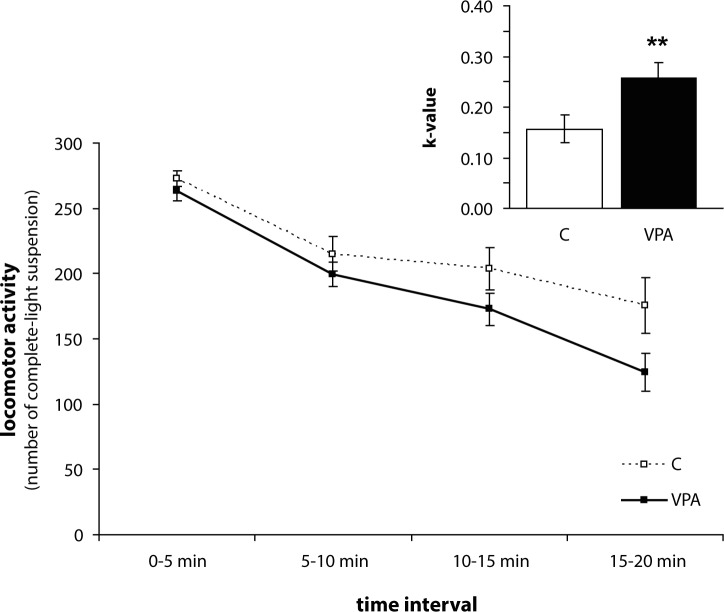
Habituation dynamics and rate (k-value) of control (C, n=18) and valproic (VPA, n=18) rats in puberty. Data are given as means ± SEM. Asterisks indicate significant differences between C and VPA groups (** *p<*0.01).

**Figure 4:**
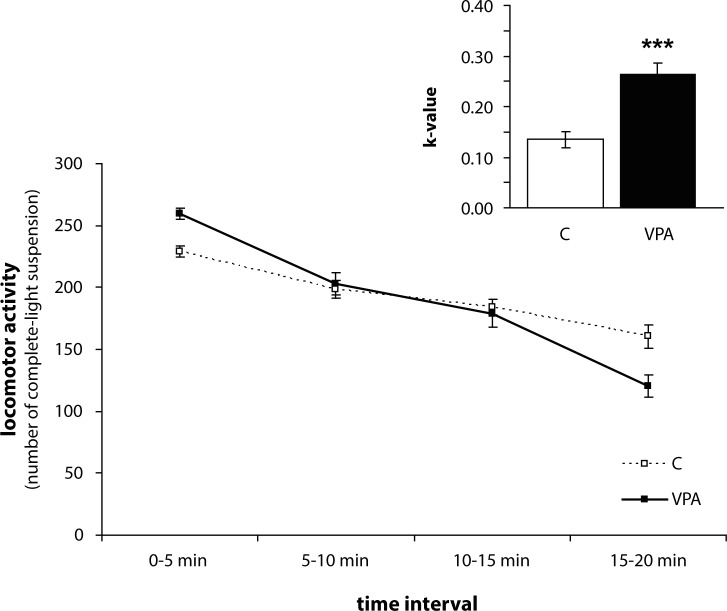
Habituation dynamics and rate (k-value) of control (C, n=18) and valproic (VPA, n=18) rats in adulthood. Data are given as means ± SEM. Asterisks indicate significant differences between C and VPA groups (*** *p<*0.01).

Statistical analysis of habituation rate (k-value) has revealed a significant effect of group (F_1,28_=8.702; *p<*0.01), age (F_2,56_=25.503; *p<*0.001), and interaction age*group (F_2,56_=7.605; *p<*0.01). Differences in habituation rate during the weaning period were not significant between VPA and C group (Figure 2). VPA rats showed a higher rate of habituation in the open-field compared to C rats in puberty (*p<*0.01) (Figure 3) and in adulthood (*p<*0.001) (Figure 4). Similarly to locomotor activity, sex differences were not found at any stage of ontogenesis.

Significant changes in k-value were observed between weaning and puberty (means ± SEM: weaning 0.01±0.03; puberty 0.27±0.03; *p<*0.001), and between weaning and adulthood (means ± SEM: weaning 0.01±0.03; adulthood 0.26±0.02; *p<*0.001) in VPA group as well as between weaning and puberty (means ± SEM: weaning 0.07±0.02; puberty 0.16±0.03; *p<*0.05) in C group.

## Discussion

Exploratory behaviour is one of the basic forms of behaviour and its prenatal disruption can reflect a CNS developmental damage. Over the last 10 years, more links have been made between autism and limited environmental exploration (O'Neil & Happé, 2000; Pierce & Courchesne, 2001).

Our study performed on an animal model of autism supports these findings. Prenatal exposure to VPA on the 12.5^th^ day of gestation had an effect on postnatal exploratory behaviour and habituation of this activity in rats during ontogeny.

When compared to the C group, VPA rats showed a lower level of locomotor component of exploratory behaviour in puberty and adulthood, but the groups did not differ at weaning. Lower exploratory behaviour of VPA rats (compared to C group) was described in puberty (Schneider & Przewłocki, 2005; Schneider *et al.*, 2006; Kerr *et al.,* 2013) and also in adulthood (Schneider & Przewłocki, 2005; Schneider *et al.*, 2006), but information about exploratory behaviour of these rats during the weaning period are missing. Reduction in exploratory behaviour in adulthood was documented in other animal models of autism – Pax6 heterozygous mutant (rSey^2^/+) rats with alterations in serotonergic system (Umeda *et al.,* 2010), mice with disruption in chromosome 7 (analogue of human locus 15q11-13) (Tamada *et al.,* 2010), BALB/cByJ mice (Moy *et al.,* 2008).

Our findings that did not show any significant differences between the quantity of locomotor activity of VPA and C groups in the weaning period support the research of Ohta *et al.* (1987) realised on autistic individuals. According to these authors, developmental motor delays, only minimally different during infancy, may become magnified with age. However, the human study of Flanagan and colleagues (2012) documented the existence of qualitative motor development differences in 6-month-aged children. Therefore, further studies are needed to reveal other potential qualitative motor as well as behavioural changes in the early development of VPA rats.

The intensity of exploratory behaviour and the locomotor part of this activity increased during adolescence (Lynn & Brown, 2009). In our study, C animals showed a significant increase of locomotor activity in puberty and adulthood compared to the weaning period. But in the VPA group, there were no significant differences between ontogenetic stages and an increase in locomotor activity during adolescence was absent. Changes in exploratory behaviour can reflect developmental damage of the CNS. One potential explanation of reduced exploratory behaviour in VPA rats may be reduced number of Purkinje cells in cerebellar vermal lobules (Rodier *et al.,* 1996, 1997). Similarly, reduced cerebellar vermal lobules, which correlated with reduction of exploration, were observed by Pierce and Courchesne (2001) in autistic children. The second potential explanation could be changes in neural structures involved in regulation of fear. This includes the medial prefrontal cortex and amygdala. Abnormalities in these structures were observed both in VPA rats (Markram *et al.,* 2008; Rinaldi *et al.,* 2008; Sui & Chen, 2012) and in autistic people (Schumann *et al.,* 2004; Bachevalier & Loveland 2006). According to Schneider and Przewłocki (2005), decreased exploration in adult VPA rats may be mediated rather by fear-related inhibition of exploratory behaviour. In an autistic population, even minor changes in the environment may induce confusion and distress, while fear of a possible change can be a further source of anxiety (Groden *et al.,* 1994; Gillott *et al.,* 2001).

However, reduction of exploration may be the result of decreased motivation to explore a novel environment (Schneider & Przewłocki, 2005), which is related with changes in neural structures involved in regulation of motivation. Decrease of motivation is one of the core symptoms of autism defined by the American Psychiatric Association (2000).

We did not observe significant changes in locomotor activity between males and females, neither within individual groups, nor in total. And yet it is well known that females should be more active than males from puberty onwards (Lynn & Brown, 2009).

The quantitative changes in exploration of an unknown environment (testing chamber) are associated with the process of habituation. Habituation is classically defined as the waning of a response, elicited by repeated exposure to a novel stimulus not accompanied by any biologically relevant consequence, either positive or negative (Leussis & Bolivar, 2006). This process reflects the neurological development of individuals and is changeable during ontogenesis. Some studies indicate that younger animals do not react as do older animals in exploratory situations (Chapillon & Roullet, 1997).

Our VPA treated rats exhibited changes in decrease of exploratory behaviour after exposure to a novel environment and habituated more rapidly than did C in puberty and adulthood. Similarly as in exploratory behaviour, we did not find any differences in the habituation process during the weaning period.

In ontogenesis, VPA rats, comparably to C, showed an increased habituation rate during adolescence. Younger animals, in both cases, showed a lower rate of habituation, which corresponds to literature information that young animals habituate more slowly than do their older counterparts (Parsons *et al.,* 1973; Chapillon & Roullet, 1997). Information about the habituation process in VPA rats have so far been absent, but in autistic patients a slower neural habituation in the amygdala and hippocampus was reported after presentation of images of faces – visual social stimulus (Kleinhans *et al.,* 2009; Webb *et al.,* 2010). In our observations, the rate of habituation, as a reaction to an unknown environmental stimulus was increased in VPA rats.

The habituation process can be affected by a variety of factors including arousal level, attention, learning, memory, and fear of novelty influencing exploratory behaviour and thus also habituation (Leussis & Bolivar, 2006).

We hypothesise that differences in the rate of habituation may be related with differences in stimulus characters and with different motivational value of this stimulus. However, the understanding of changes occurring in habituation requires further studies, aimed especially on discovering neural mechanisms participating in the regulation of this process in VPA rats.

VPA rats show many features characteristic for the autistic population, but in our opinion these findings call for further research. In our article we mentioned only behavioural changes in VPA rats but we will continue in deciphering neural mechanism associated with regulation of the specific types of behaviour observed in these animals.

The questions why behavioural changes were discovered in puberty and adulthood and why we did not observe intersex differences in contrast to other authors, remain still open.

## Conclusion

Our behavioural study supports the findings of developmental changes in the nervous system of VPA rats. These changes are not only demonstrating as changes in locomotor activity but also as so far unpublished changes in the process of habituation. We believe that these findings will contribute to the validity of the model to improve research of autism.
